# N‐glycome inheritance from cells to extracellular vesicles in B16 melanomas

**DOI:** 10.1002/1873-3468.13377

**Published:** 2019-04-11

**Authors:** Yoichiro Harada, Yasuhiko Kizuka, Yuko Tokoro, Kiyotaka Kondo, Hirokazu Yagi, Koichi Kato, Hiromasa Inoue, Naoyuki Taniguchi, Ikuro Maruyama

**Affiliations:** ^1^ Department of Systems Biology in Thromboregulation Kagoshima University Graduate School of Medical and Dental Sciences Japan; ^2^ Center for Highly Advanced Integration of Nano and Life Sciences (G‐CHAIN) Gifu University Japan; ^3^ Department of Pulmonary Medicine Kagoshima University Graduate School of Medical and Dental Sciences Japan; ^4^ Graduate School of Pharmaceutical Sciences Nagoya City University Japan; ^5^ Exploratory Research Center on Life and Living Systems (ExCELLS) Institute for Molecular Science (IMS) National Institutes of Natural Sciences Okazaki Japan; ^6^ Department of Glyco‐Oncology Osaka International Cancer Institute Japan

**Keywords:** asparagine‐linked glycans, extracellular vesicles, metastasis

## Abstract

We investigated the correlation between metastatic behaviors of tumor cells and asparagine‐linked glycosylation (N‐glycosylation) of tumor‐derived extracellular vesicles (EVs). Three mouse melanoma B16 variants with distinct metastatic potentials show similar gene expression levels and enzymatic activities of glycosyltransferases involved in N‐glycosylation. All melanoma variants and EVs have nearly identical profiles of de‐sialylated N‐glycans. The major de‐sialylated N‐glycan structures of cells and EVs are core‐fucosylated, tetra‐antennary N‐glycans with β1,6‐*N*‐acetylglucosamine branches. A few N‐glycans are extended by *N*‐acetyllactosamine repeats. Sialylation of these N‐glycans may generate cell‐type‐specific N‐glycomes on EVs. Taken together, melanoma‐derived EVs show high expression of tumor‐associated N‐glycans, and the core structure profile is inherited during multiple selection cycles of B16 melanomas and from tumor cells to EVs.

## Abbreviations

**EVs**, extracellular vesicles

**PA**, 2‐aminopyridine

**Figure 1:**
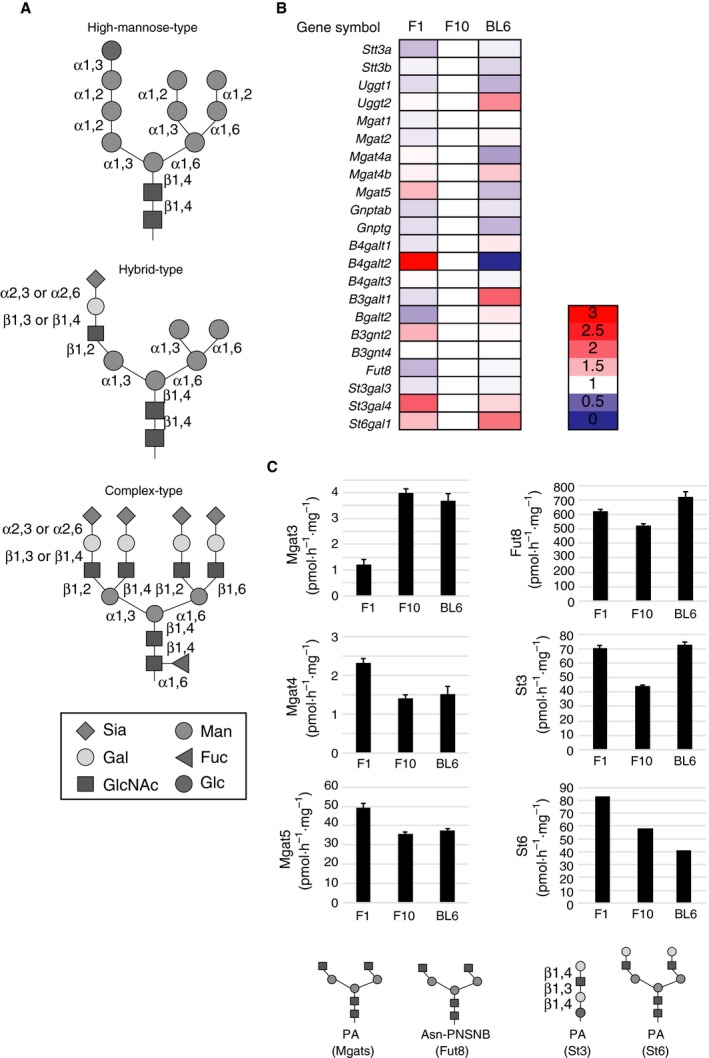
Gene expression and enzymatic activities of glycosyltransferases involved in N‐glycosylation in B16 variants. (A) Models of high‐mannose‐type, hybrid‐type, and complex‐type glycans. Sia, sialic acid; Gal, galactose; GlcNAc, *N*‐acetylglucosamine; Man, mannose; Fuc, fucose; Glc, glucose. (B) Relative gene expression levels of 22 glycosyltransferases involved in N‐glycosylation in B16 variants. Gene expression levels in B16‐F10 cells were set to 1.0. The values were calculated as the means of two independent experiments. (C) Enzymatic activities of Mgat3, Mgat4, Mgat5, Fut8, α2,3‐sialyltransferase (St3) and α2,6‐sialyltransferase (St6) in B16 variants. Data represent means ± standard errors from four independent experiments (for Mgats, Fut8 and St3). Activity assay for St6 was performed once due to limited availability of the acceptor substrate. 2‐Aminopyridine (PA)‐labeled acceptor glycans used for enzyme activity assays were shown. PNSNB,* N*‐(2‐(2‐pyridylamino)ethyl)succinamic acid 5‐norbor‐ nene‐2,3‐dicarboxyimide ester.

Most cell surface and secretory proteins are modified by asparagine‐linked glycans (N‐glycans) 1, which play indispensable roles in regulation of protein quality control, intracellular trafficking, and cell‐to‐cell communication. Based on their monosaccharide compositions, N‐glycans are classified into high‐mannose‐type, hybrid‐type, and complex‐type glycans (Fig. 1A). The distal ends of complex‐type glycans are typically capped with sialic acid (Sia) 2 and the innermost *N*‐acetylglucosamine (GlcNAc) may be fucosylated (core fucosylation) 3, 4. In tumor cells, N‐glycan structures frequently become more extensively core‐fucosylated, more highly branched, and hyper‐sialylated (Fig. 1A) 5, 6, 7. All of these glycosylation alterations associated with malignant transformation can promote tumor progression and metastasis 8, 9, 10.

Blood‐borne tumor metastasis is a complex process involving tumor cell invasion into normal tissues, intravasation to the circulation, extravasation, and colonization at distant organs 11. To investigate these processes, multiple *in vivo* and *in vitro* selections have been performed to obtain mouse B16 malignant melanoma variants with distinct metastatic potentials. The B16‐F1 (poorly lung‐colonizing) and B16‐F10 (highly lung‐colonizing) variants are selected for their lung colonization abilities following intravenous injection of the parent B16 cells (experimental metastasis) 12. These variants show little spontaneous metastasis from subcutaneous tumors to the lungs 13. Meanwhile, the B16‐BL6 variant is selected for its high invasive ability following injection of the parent B16‐F10 variant into the urinary bladder *in vitro*, resulting in a highly spontaneous metastatic variant 13.

Tumor cells secrete small vesicles, termed extracellular vesicles (EVs), that contain various cargo molecules including nucleic acids, soluble proteins, and membrane proteins 14. Tumor‐derived EVs promote tumor progression and metastasis by delivering their cargo molecules to surrounding tumor microenvironments and future metastatic organs 14. Accumulating evidence has demonstrated that the molecular compositions of tumor‐derived EVs are dependent on the metastatic potentials of their secreting tumor cells 15, 16, 17, suggesting a potential role of tumor‐derived EVs as biomarkers for metastasis. Although aberrant N‐glycosylation on tumor cells is known to promote tumor metastasis 6, little is known about the correlation between N‐glycosylation of tumor‐derived EVs and metastatic potentials of the secreting tumor cells.

In the present study, we investigated the correlation between metastatic potentials of three B16 variants (B16‐F1, B16‐F10, B16‐BL6) and N‐glycosylation of their EVs by characterizing N‐glycan structures and gene expression, as well as enzymatic activity, of glycosyltransferases involved in N‐glycosylation. The results demonstrate that the core structure profile of N‐glycans is inherited at the genetic level during multiple *in vivo* and *in vitro* selection cycles of B16 variants and is copied from tumor cells to their EVs. It was suggested that sialylation of these N‐glycans probably generates cell‐type‐specific N‐glycome on EVs. This study establishes the N‐glycosylation landscapes of B16 variants and their EVs, and indicates that the bulk N‐glycosylation of melanoma EVs does not reflect the metastatic potentials of their secreting tumor cells in the B16 model.

## Materials and methods

### Cell culture

Mouse B16‐F1 and B16‐F10 melanoma cells were purchased from the American Type Culture Collection (Manassas, VA, USA). B16‐BL6 cells were purchased from RIKEN Bioresource Center. All cell lines were maintained in complete Dulbecco's modified Eagle's medium containing 10% fetal bovine serum, 100 U·mL^−1^ penicillin, and 100 μg·mL^−1^ streptomycin at 37 °C in a 5% CO_2_ atmosphere.

### Preparation of EVs

Extracellular vesicles were prepared as described 18. Briefly, melanoma cells (1 × 10^6^ cells/10‐cm dish) were cultured for 24 h, washed twice with PBS, and incubated for 48 h in EV‐depleted medium. The conditioned medium was sequentially centrifuged at 130 ***g*** for 5 min, 20 000 ***g*** for 20 min, and 100 000 ***g*** for 70 min. The final pellet containing EVs was washed once with PBS and measured for its protein concentration with a BCA protein assay kit (Pierce, Rockford, IL, USA), using bovine serum albumin as an external standard.

### Preparation of whole cell lysates

Cells were lysed for 15 min at 0 °C in lysis buffer comprising 10 mm Tris‐HCl (pH 7.4), 150 mm NaCl, 1% Triton X‐100, 1 mm EDTA, and protease inhibitor cocktail (Roche, Mannheim, Germany). The homogenate was centrifuged at 20 000 ***g*** for 10 min and the supernatant was recovered as the whole cell lysate.

### Preparation and fluorescent labeling of N‐glycans

N‐glycans were released from EVs and whole cell lysates by treatment with peptide‐*N* ^4^‐(*N*‐acetyl‐β‐glucosaminyl)asparagine amidase F (PNGase F) (New England Biolabs, Ipswich, MA, USA) according to the manufacturer's instructions. The released N‐glycans were purified and fluorescently labeled with 2‐aminopyridine (PA) using a BlotGlyco Kit (Sumitomo Bakelite, Tokyo, Japan) according to the manufacturer's instructions.

### HPLC

PA‐labeled N‐glycans were initially separated into one neutral fraction and six sialidase‐sensitive fractions by anion‐exchange HPLC, as described 19. Sialidase sensitivity was assessed by incubating the anion‐exchange HPLC fractions with 10 mU neuraminidase from *Arthrobacter ureafaciens* in 10 mm sodium acetate buffer (pH 5.5) for 16 h at 37 °C. The sialidase‐digested samples and the neutral fraction were desalted on PD‐10 desalting columns (GE Healthcare, Chicago, IL, USA) and subjected to reversed‐phase HPLC as described 19. The major peaks (> 0.5% of total glycans) from the reversed‐phase HPLC were further fractionated and analyzed by size‐fractionation HPLC to determine the glycan structures and quantify their abundance using PA‐labeled glucose hexamers in PA‐glucose oligomers (degree of polymerization = 3–22; 2 pmol·μL^−1^; Takara), as described 19.

### Mass spectrometric analysis of PA‐labeled N‐glycans

PA‐labeled N‐glycans fractionated by reversed‐phase HPLC were subjected to matrix‐assisted laser desorption/ionization time‐of‐flight mass spectrometric analysis. PA‐glycans (1 μL) were mixed with 1 μL of 2,5‐dihydroxybenzoic acid (10 mg/mL in 50% acetonitrile/0.1% trifluoroacetic acid) on a target plate. After evaporation to dryness, spectra were obtained in the positive mode using an Autoflex mass spectrometer (Bruker Daltonics) operated in the reflector mode.

### Structural determination of PA‐labeled N‐glycans from melanoma EVs

The structures of PA‐labeled N‐glycans from melanoma EVs were determined by reference to a database, glycoanalysis by the three axes of MS and chromatography (GALAXY) 20 version 2 (http://www.glycoanalysis.info/galaxy2/ENG/index.jsp), using the masses and glucose units of individual glycans, the latter of which were calculated by the elution positions of PA‐glucose oligomers (degree of polymerization = 3–22) in the reversed‐phase and size‐fractionation chromatography 19. The glycan structures were validated by analyzing samples in parallel with standard PA‐labeled N‐glycans (code numbers: 200.4, 210.13, 210.4, 300.22, 300.8, 310.18, 310.19, 310.8, 400.22, 410.16, 410.22, 410.42) in GALAXY 20 by reversed‐phase HPLC.

### Western blot analysis

Samples (10 μg protein) were denatured for 3 min at 100 °C, separated by SDS/PAGE, and analyzed by western blotting using an anti‐CD81 antibody (1 : 5000; B‐11; Santa Cruz Biotechnology, Santa Cruz, CA, USA).

### Glycosyltransferase gene expression

Real‐time PCR for glycosyltransferases was performed as described previously 21. Briefly, total RNA was extracted from mouse melanoma cells (1 × 10^6^ cells/10‐cm dish) that had been cultivated for 48 h using TRI Reagent (Molecular Research Center, Inc., Cincinnati, OH, USA) in accordance with the manufacturer's protocol, followed by treatment with DNase (Qiagen) and purification using RNeasy Mini kit (Qiagen, Hilden, Germany). 1 μg total RNA was reverse‐transcribed using an R^2^ First Strand Kit (Qiagen) in a 40‐μL reaction mixture and then diluted with 182 μL RNase‐free water. The cDNA thus obtained was mixed with 2.7 mL RT2 SYBR Green qPCR Mastermix (Qiagen) and 2.496 mL water, and 25 μL of the mixture was applied to each well of a 96‐well plate that contained specific primers for glycotransferase mRNAs (Qiagen). Two types of 96‐well plates were used for quantification of 144 glycosyltransferase genes; a commercially available plate for Mouse Glycosylation (Cat. No. 330231 PAMM‐046ZA) covering 84 glyco‐related genes, and a 96‐Well Custom PCR Array in which we manually selected 86 glycosyltransferase genes, seven house keeping genes (*Actb*,* Gapdh*,* Hsp90a*,* Pgk1*,* B2m*,* Gusb*,* Ldha*) and three primers for monitoring genomic contamination, cDNA synthesis and PCR reaction. cDNAs were amplified and analyzed using an ABI PRISM 7900HT thermocycler (Applied Biosystems, Waltham, MA, USA) according to the RT^2^ Profiler PCR Array Handbook (Qiagen). The abundance of glycotransferase mRNAs relative to that of housekeeping genes (the average of *Actb*,* B2m*,* Gapdh* and *Hsp90ab1*) was calculated using the Δ*C*_t_ method. The values in Tables 1 and Table S1 were calculated as the means of two independent experiments.

**Table 1:** mRNA abundances of N‐glycosylation‐related glycosyltransferases relative to the mean abundance of four housekeeping genes (*Actb*,* B2m*,* Gapdh*, and *Hsp90ab1*) in B16 variants. Fuc‐T, fucosyltransferase; Gal‐T, galactosyltransferase; GlcNAc‐1‐P‐T, GlcNAc‐1‐phosphate transferase; GlcNAc‐T, GlcNAc transferase; Glc‐T, glucosyltransferase; ND, not detected (mRNA abundance less than 0.001); OST, oligosaccharyltransferase; Sia‐T, sialyltransferase

Gene symbol	Category	mRNA abundance		BL6
		F1	F10	
*Stt3a*	OST subunit	0.418	0.543	0.505
*Stt3b*	OST subunit	0.347	0.368	0.313
*Uggt1*	α1,3Glc‐T	0.087	0.100	0.074
*Uggt2*	α1,3Glc‐T	0.017	0.016	0.028
*Mgat1*	β1,2GlcNAc‐T	0.014	0.015	0.015
*Mgat2*	β1,2GlcNAc‐T	0.011	0.012	0.013
*Mgat4a*	β1,4GlcNAc‐T	0.071	0.066	0.044
*Mgat4b*	β1,4GlcNAc‐T	0.164	0.146	0.200
*Mgat5*	β1,6GlcNAc‐T	0.180	0.123	0.095
*Gnptab*	GlcNAc‐1‐P‐T	0.052	0.061	0.055
*Gnptg*	GlcNAc‐1‐P‐T	0.014	0.016	0.012
*B4galt1*	β1,4Gal‐T	0.027	0.030	0.035
*B4galt2*	β1,4Gal‐T	0.003	0.001	ND
*B4galt3*	β1,4Gal‐T	0.020	0.019	0.018
*B3galt1*	β1,3Gal‐T	0.007	0.008	0.016
*B3galt2*	β1,3Gal‐T	0.004	0.006	0.007
*B3gnt2*	β1,3GlcNAc‐T (polylactosamine)	0.027	0.018	0.019
*B3gnt4*	β1,3GlcNAc‐T (polylactosamine)	0.001	0.001	0.001
*Fut8*	α1,6Fuc‐T (core fucose)	0.021	0.028	0.027
*St3gal3*	α2,3Sia‐T	0.019	0.021	0.020
*St3gal4*	α2,3Sia‐T	0.083	0.041	0.053
*St6gal1*	α2,6Sia‐T	0.026	0.018	0.034

### Glycosyltransferase activity assay

Enzymatic activity of mannosyl (β1,4‐)‐glycoprotein β1,4‐GlcNAc transferase (Mgat3), α‐1,3‐mannosyl‐glycoprotein 4‐β‐GlcNAc transferase (Mgat4), α‐1,6‐mannosylglycoprotein 6‐β‐GlcNAc transferase (Mgat5), fucosyltransferase 8 (Fut8), α2,3‐sialyltransferase and α2,6‐sialyltransferase were measured as described previously 22, 23, 24 with modifications. Cells were lysed with tris‐buffered saline containing 1% Nonidet P‐40 and a protease inhibitor cocktail (Roche), and the lysates were directly used as enzyme sources. As positive controls, N‐terminal His‐tagged truncated human MGAT3, mouse Mgat4a, human MGAT5, human FUT8, human ST3GAL4 and human ST6GAL1 were expressed in COS‐7 cells and purified from culture media (see below). To prepare GGnGGnbi‐PA (β1,4‐galactosylated form of GnGnbi‐PA) as an acceptor substrate for α2,6‐sialyltransferase, GnGnbi‐PA was incubated with purified protein A‐B4GALT1 22 in 125 mm MES pH 6.2, 10 mm MnCl_2_, 10 mm UDP‐Gal at 37 °C overnight. The enzymes were mixed with various concentrations of a fluorescence‐labeled acceptor substrate (GnGnbi‐PA 23 for Mgats, GnGnbiAsn‐PNSNB 23 for FUT8, LNnT‐PA 22 for α2,3‐sialyltransferase and GGnGGnbi‐PA for α2,6‐sialyltransferase) in 10 μL of a reaction buffer containing 125 mm MES pH 6.2, 10 mm MnCl_2_, 0.2 m GlcNAc, 0.5% Triton X‐100, 1 mg·mL^−1^ bovine serum albumin and donor substrates, followed by incubation at 37 °C. As donor substrates, 20 mm UDP‐GlcNAc, 1 mm GDP‐Fuc, and 1 mm CMP‐*N*‐acetylneuraminic acid were used for Mgat, fucosyltransferase and sialyltransferase activity assays, respectively. After boiling for 5 min to stop reaction, 40 μL of water was added, followed by centrifugation at 15 000 ***g*** for 5 min. The supernatant was analyzed by reverse‐phase HPLC (Prominence, Shimadzu) equipped with an ODS column (TSKgel ODS‐80TM, TOSOH Bioscience, Tokyo, Japan) 22, 23, 24.

### Expression and purification of recombinant glycosyltransferases

cDNAs encoding the catalytic regions of human MGAT3, mouse Magt4a, human MGAT5, human FUT8, human ST3GAL4 and human ST6GAL1 were amplified by PCR with primers: human MGAT3, 5′CGGAATTCGAGCCAGGAGGCCCTGACCT3′ and 5′CTAGACTTCCGCCTCGTCCA3′; mouse Mgat4a, 5′CGGAGCTAAACACCATTGTC3′ and 5′GTGCTCGAGTCTAAGCAGATCAACTGGTG3′; human MGAT5, 5′CTACAGCTGTTCCCAGCTTG3′ and 5′AGGCTCGAGCTATAGGCAGTCTTTGCAGA3′; human FUT8, 5′CGGAATTCCGGATACCAGAAGGCCCTAT3′ and 5′TTATTTCTCAGCCTCAGGAT3′; human ST3GAL4, 5′GAGGAATTCGAGCCGTGCCTCCAGGGTGA3′ and 5′TTGCTCGAGTCAGAAGGACGTGAGGTTCTTG3′; and human ST6GAL1, 5′GTGGAATTCAAGGACAGCTCTTCCAAAAA3′ and 5′AAACTCGAGTTAGCAGTGAATGGTCCGGAA3′.

The PCR products were inserted into pcDNA‐IH as described previously 23, 24. COS7 cells were transfected with the plasmids, and the media were replaced with Opti‐MEM I, followed by incubation for 3 days. The truncated N‐terminally His‐tagged enzymes secreted into the media were purified through a Ni^2^+‐column and desalted with NAP‐5 gel filtration column (GE Healthcare).

## Results and Discussion

### Glycosyltransferase gene expression landscapes in B16 variants

The cellular N‐glycome is regulated by multiple factors including the expression profiles of glycosyltransferase genes 25. To clarify the gene expression landscapes in three B16 variants with distinct metastatic potentials (B16‐F1, B16‐F10, B16‐BL6), the expression levels of 144 glycosyltransferase genes were analyzed by real‐time PCR (Fig. S1 and Table S1) and those involved in N‐glycosylation were shown in Fig. 1A and Table 1. In B16‐F10 cells, we detected the expression of 22 genes that are involved in N‐glycosylation reaction (*Stt3a* and *Stt3b*), protein quality control in the ER (*Uggt1* and *Uggt2*), GlcNAc branch formation (*Mgat1*,* Mgat2*,* Mgat4a*,* Mgat4b* and *Mgat5*), lysosome targeting (*Gnptab* and *Gnptg*), galactosyltransferases (*B4galt1‐3*,* B3galt1* and *B3galt2*), polylactosamine formation (*B3gnt2* and *B3gnt4*), core fucosylation (*Fut8*), α2,3‐sialyltransferases (*St3gal3* and *St3gal4*) and α2,6‐sialyltransferase (*St6gal1*). The gene expression pattern of B16‐F1 and B16‐BL6 cells was similar to that of B16‐F10 cells, except that *B4galt2* was undetectable in B16‐BL6 cells. We hardly detected expression of *Mgat3* (bisecting GlcNAc formation), as well as *Fut1‐7* and *Fut9* (H, Se, Lewis, sialyl Lewis^x^ antigen formation), in all three variants. In comparison of cells with different lung colonization ability (B16‐F1 and B16‐F10), B16‐F1 cells showed higher expression of *Mgat5* (1.5‐fold), *B4galt2* (3‐fold), *B3gnt2* (1.5‐fold), *St3gal4* (2.0‐fold) and *St6gal1* (1.4‐fold) than B16‐F10 cells. In contrast, B16‐F1 cells expressed lower levels of *Stt3a* (0.8‐fold), *B3galt2* (0.7‐fold) and *Fut8* (0.8‐fold) than B16‐F10 cells. In comparison of cells with different invasiveness (B16‐F10 and B16‐BL6), B16‐BL6 cells showed higher levels of *Uggt2* (1.8‐fold), *Mgat4b* (1.4‐fold), *B4galt1* (1.2‐fold), *B3galt2* (1.2‐fold), *St3gal4* (1.3‐fold) and *St6gal1* (1.9‐fold) than B16‐F10 cells. In contrast, B16‐BL6 cells showed lower expression of *Uggt1* (0.7‐fold), *Mgat4a* (0.7‐fold) and *Mgat5* (0.8‐fold) than B16‐F10 cells, suggesting that low expression of *Uggt1* and *Mgat4a* is compensated by high expression of *Uggt2* and *Mgat4b* in B16‐BL6 cells. Together, these results indicate that B16 variants with distinct metastatic potential have unique glycosyltransferase gene expression patterns. However, overall N‐glycosylation‐related gene profiles are inherited during multiple selection cycles of B16 variants.

### Glycosyltransferase activities in B16 variants

To correlate glycosyltransferase gene expression profiles and the enzymatic activities, we measured activities of Mgat3, Mgat4, Mgat5, Fut8, α2,3‐and α2,6‐sialyltransferases by using fluorescently labeled acceptor glycans (Fig. 1C). Consistent with the fact that *Mgat3* gene was rarely expressed in all three B16 variants (Table S1), the enzymatic activity was found to be very low. Among B16 variants, B16‐F1 cells showed the lowest Mgat3 activity, while this variant had higher Mgat4 and Mgat5 activities than B16‐F10 and B16‐BL6 cells. It is well known that Mgat5 is deeply involved in cancer metastasis 24 and that exogenous expression of Mgat3 inhibits experimental lung metastasis in murine model 26 because bisecting GlcNAc formed by Mgat3 has steric hindrance effects on the action of Mgat5 27. However, Mgat5 activity in B16 variants was very high, suggesting that basal levels of Mgat3 activity do not inhibit the action of Mgat5 in the cells. The Fut8 activity was high in B16‐BL6 cells as compared with the other two variants. The α2,3‐sialyltransferase activity was low in B16‐F10 cells and similar between B16‐F1 and B16‐BL6 variants. The degree of enzymatic activity of Mgat3, Mgat4, Mgat5, Fut8 and α2,3‐sialyltransferase (Fig. 1C) was in good agreement with their gene expression levels in B16 variants (Fig. 1B). However, α2,6‐sialyltransferase activity in B16 variants was not associated with the gene expression pattern of *St6gal1* and decreased over selection cycles. Together, these results indicate that B16 variants show unique glycosyltransferse profiles and imply that B16 variants have cell‐type‐specific N‐glycome.

### Identification of N‐glycan structures of EVs from B16‐F10 cells

**Figure 2:**
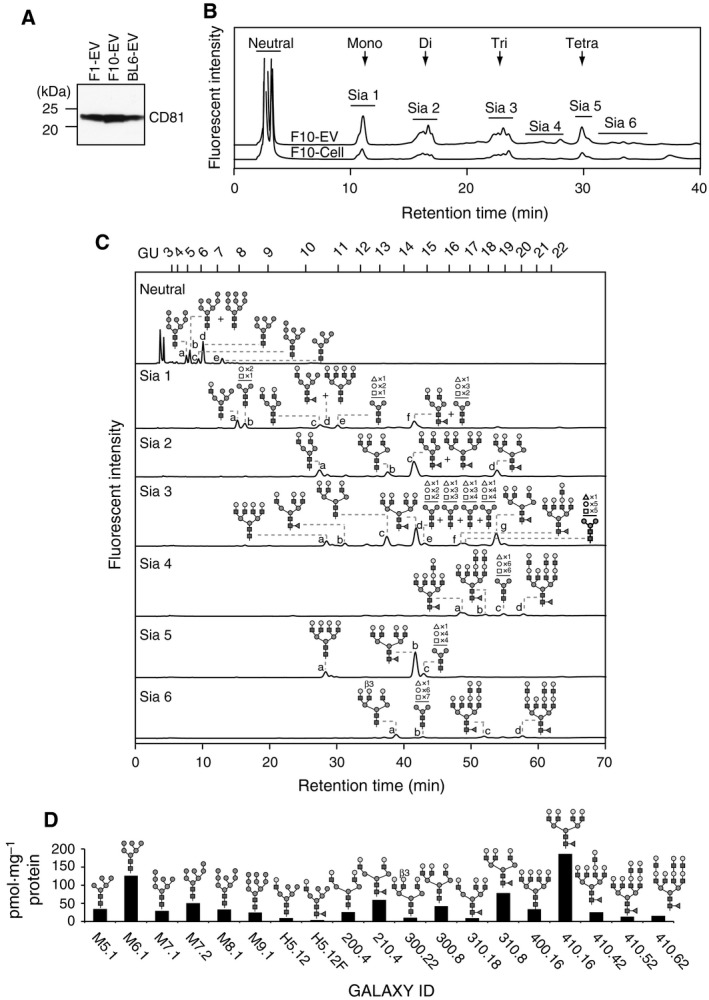
Identification of N‐glycan structures expressed on EVs from the B16‐F10 variant. (A) Western blot analysis of EVs from B16 variants (F1‐EV, F10‐EV, BL6‐EV) using an anti‐CD81 antibody. (B) Anion‐exchange chromatography of fluorescently labeled N‐glycans prepared from B16‐F10 cells (F10‐Cell, 2.5 mg protein/injection) and F10‐EV (1.4 mg protein/injection). Neutral, unbound fraction; S1–S6, fractions containing sialylated N‐glycans. The positions of mono‐, di‐, tri‐, and tetra‐sialylated N‐glycans were determined as described 33. (C) The fractions from anion‐exchange chromatography of F10‐EVs were isolated, de‐sialylated, and analyzed by reversed‐phase HPLC. GU, glucose units based on elution positions of standard glucose oligomers. (D) Quantification of N‐glycans expressed on F10‐EVs after size‐fractionation chromatography. The identity (ID) for each N‐glycan structure was assigned based on the GALAXY database version 2.

To determine N‐glycosylation profiles of EVs from B16 variants, they were prepared from the conditioned media by differential centrifugation. Consistent with our previous study on the extensive biochemical characterization of B16‐F10‐derived EVs (F10‐EVs) 18, the EV preparation contained an EV marker protein CD81 (Fig. 2A). F1‐EVs and BL6‐EVs were also prepared by the same procedures and found to express CD81 (Fig. 2A). To compare the yield of EVs, we seeded the same number of cells in the same volume of culture medium, and obtained EVs from B16‐F1 cells (1307 ± 465 μg protein), B16‐F10 cells (1258 ± 140 μg protein) and B16‐BL6 cells (578 ± 62 μg protein).

Among the three B16 variants, the B16‐F10 variant is positioned in the middle of the selection cycles 12, 13. Thus, we used this variant for extensive N‐glycan analysis. N‐glycans were released from F10‐EVs by PNGase F digestion, fluorescently labeled, and separated into one neutral and six sialidase‐sensitive fractions by anion‐exchange HPLC (Fig. 2B). The number of sialic acids present on N‐glycans of F10‐EVs was similar to that of B16‐F10 cells. These fractions were collected and analyzed by reversed‐phase HPLC, revealing similar overall elution profiles between B16‐F10 cells and F10‐EVs (Fig. S2). However, some sialylated N‐glycans in the Sia 2, Sia 3, Sia 4 and Sia 6 fractions were more enriched in F10‐EVs than B16‐F10 cells (Fig. S3), suggesting that sialylation probably generates EV‐specific N‐glycome.

As the sialylation of N‐glycans generated considerable structural heterogeneity, de‐sialylated N‐glycans were subjected to further structural analyses. Neutral and de‐sialylated Sia 1‐6 fractions from anion‐exchange HPLC were further fractionated by reversed‐phase HPLC (Fig. 2C) and subjected to size‐fractionation HPLC and mass spectrometry to determine the glycan structures by the GALAXY database and quantify their abundance (Fig. 2D and Table S2). We detected 29 N‐glycans and reliably deduced 19 structures. The relative amounts of complex‐type, hybrid‐type, and high‐mannose‐type glycans were found to be 62%, 1%, and 37%, respectively. Of these, core‐fucosylated, tetra‐antennary glycans with GlcNAc branches formed by Mgat1 (GlcNAcβ1,2‐Manα1,3‐), Mgat2 (GlcNAcβ1,2‐Manα1,6‐), Mgat4 (GlcNAcβ1,4‐Manα1,3‐), and Mgat5 (GlcNAcβ1,6‐Manα1,6‐) (Fig. 2D, GALAXY ID: 410.16) occupied a large fraction of the N‐glycans of F10‐EVs. The galactose residues at the nonreducing end of N‐glycans can be extended by *N*‐acetyllactosamine repeats (polylactosamine), which can serve as ligands for galactose‐binding lectins (galectins) 28 and promote tumor progression 29. Small, but significant, amounts of polylactosamine‐bearing N‐glycans were detected in the Sia 3, Sia 4, and Sia 6 fractions of F10‐EVs (Fig. 2C,D; GALAXY ID: 410.42, 410.52 and 410.62). Taken together, our data demonstrate that F10‐EVs contain tumor‐associated N‐glycans.

### Inheritance of core structure profiles of N‐glycans during selection cycles of B16 variants and from the variants to their EVs

**Figure 3:**
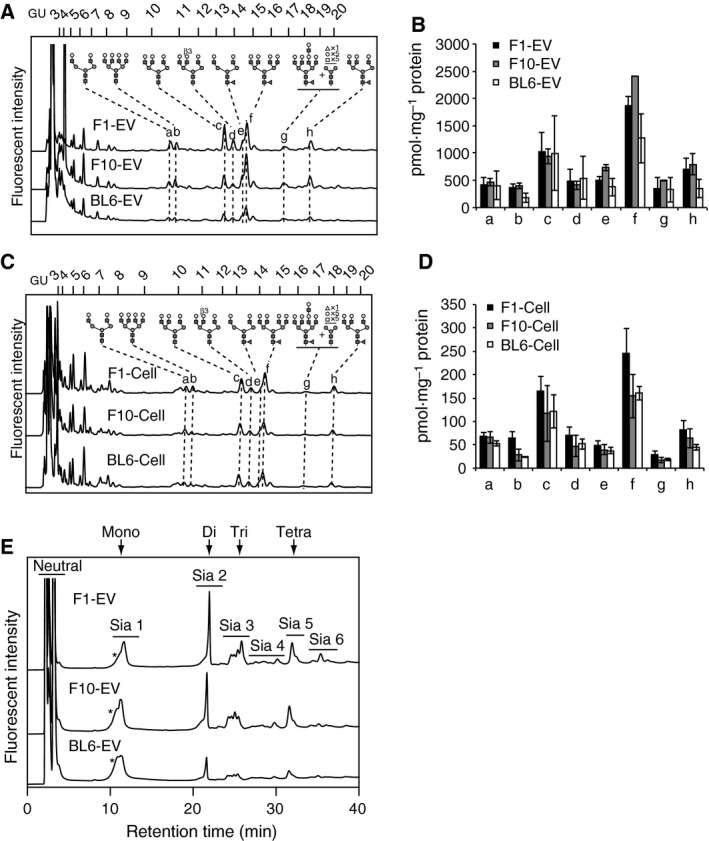
Comparison of N‐glycans between B16 variants and their EVs. (A–D) N‐glycans from EVs (A and B; F1‐EV, F10‐EV, BL6‐EV) and B16 variants (C and D; F1‐Cell, F10‐Cell, BL6‐Cell) were de‐sialylated and directly analyzed by reversed‐phase chromatography. GU, glucose units based on elution positions of standard glucose oligomers. The amounts of peaks a–h were estimated based on those of PA‐glucose hexamers (B and D). Data represent means ± standard deviations from three independent experiments. (E) Anion‐exchange chromatography of N‐glycans from EVs (F1‐EV, F10‐EV, BL6‐EV).

To investigate whether characteristic core N‐glycan structures were enriched in EVs, we compared reversed‐phase HPLC profiles of de‐sialylated complex‐type glycans between F10‐EVs and B16‐F10 cells. For the comparison, we chose eight major peaks that contained nine N‐glycans (Fig. 3A) and found that while the amounts of each N‐glycan in F10‐EVs were approximately 10‐fold larger than those in B16‐F10 cells, the N‐glycan profiles were very similar (Fig. 3A–D, F10‐EVs and F10‐Cells). These findings indicate that the core N‐glycan structures and their relative abundance are inherited from B16‐F10 cells to F10‐EVs.

Next, we investigated whether the N‐glycosylation profiles of EVs differed among the B16 variants. Anion‐exchange HPLC analysis of sialylated glycans obtained from F1‐EVs, F10‐EVs, and BL6‐EVs revealed that although the number of sialic acids present on N‐glycans was similar among the three variants, the Sia 3 fraction showed distinct elution patterns (Fig. 3E). Inconsistent HPLC profiles of the Sia 2 fraction between Figs 1C and 2E occurred for unknown reasons. The core structures of de‐sialylated N‐glycans showed no marked differences among the three EVs (Fig. 3A–D).

We examined whether core structure profiles of N‐glycans were inherited from B16‐F1 and B16‐BL6 variants to their EVs, and found that F1‐EVs and BL6‐EVs had nearly identical N‐glycosylation profiles to their secreting tumor cells, except that slightly higher expression of tetra‐antennary glycans in B16‐F1 cells than the other variants was not inherited to the EVs (Fig. 3A–D). The high expression of tetra‐antennary glycans in B16‐F1 cells was predictable from higher expression of Mgat4 and Mgat5, and lower expression of Mgat3, the enzyme of which is known to inhibit the action of Mgat5 26, 30, than the other two variants (Fig. 1B,C). Together, these findings indicate that the core structure profiles of N‐glycans are maintained during multiple *in vivo* and *in vitro* selection cycles of B16 variants, and mostly inherited from tumor cells to their EVs during EV generation. Our data also imply that sialylation probably generates cell‐type‐specific N‐glycome on EVs.

## Conclusion

The results of the present study provide the first detailed structural and quantitative comparisons of N‐glycans expressed in EVs and their secreting tumor cells using three B16 variants with distinct metastatic potentials. Although B16 variants showed unique profiles of gene expression and enzymatic activity of glycosyltransferases involved in N‐glycosylation, the overall core structure profiles of N‐glycans were similar between the variants. Given the critical roles of sialylation in tumor progression, it is interesting to note that B16 variants expressed the same set of sialyltransferase genes involved in N‐glycan modification. However, our data suggested that sialylation of N‐glycans probably generates unique N‐glycome in B16 variants and the EVs. Further extensive studies will be needed to elucidate the precise sialylation patterns of tumor‐derived EVs and their roles in tumor metastasis.

It was shown that B16‐F1 and B16‐F10 cells both require N‐glycosylation for adhesion to endothelial cells, as well as experimental lung metastasis 31, 32. Interestingly, the highly metastatic B16‐BL6 variant had similar N‐glycosylation profiles to the other variants, implying a general pathological role of this post‐translational modification in the establishment of lung metastasis. Experimental lung metastasis was reported to be promoted by EVs from highly metastatic melanoma cells and this EV function was clearly dependent on expression of hepatocyte growth factor receptor (Met) 15. Although Met is an N‐glycosylated protein, EVs from poorly and highly metastatic B16 variants shared the same core N‐glycosylation pattern, indicating that the bulk N‐glycosylation of melanoma‐derived EVs does not reflect the metastatic potentials of their secreting tumor cells.

In conclusion, this study establishes the N‐glycosylation landscapes of tumor‐derived EVs and their secreting tumor cells in B16 models, enabling further exploration of the functions of the N‐glycans on tumor‐derived EVs in future studies.

## Author contributions

YH designed and conducted the research. YH, YK, YT and KKondo performed the experiments. HY and KKato contributed the new reagents. YH, YK, HI, NT and IM analyzed the data. YH wrote the paper. All authors reviewed the paper.

## Funding

This work was partly supported by grants from the SENSHIN Medical Research Foundation (YH), the Kodama Memorial Fund for Medical Research (YH), and MEXT/JSPS Grants‐in‐Aid for Scientific Research (JP17H06414 to HY and 17K07356 to YK).

## Supporting information


**Fig. S1.** Relative gene expression levels of 144 glycosyltransferases in B16 variants.

**Fig. S2.** Comparative analysis of sialylated N‐glycans from B16‐F10 cells and F10‐EVs.

**Fig. S3.** Relative amounts of sialylated N‐glycans in the Sia 1‐6 fractions of B16‐F10 cells and F10‐EVs.

**Table S1.** mRNA abundances of glycosyltransferases relative to the mean abundance of four housekeeping genes (*Actb*,* B2m*,* Gapdh*, and *Hsp90ab1*) in B16 variants.
**Table S2.** Structural analysis of N‐glycans expressed on EVs from B16‐F10 cells.
