# Supplementary figures and images for: N‐glycome inheritance from cells to extracellular vesicles in B16 melanomas
Source: FEBS Lett. 2019 Apr 11;593(9):942–51. doi: 10.1002/1873-3468.13377 (PMC6594130; doi:10.1002/1873-3468.13377)

Figure S1

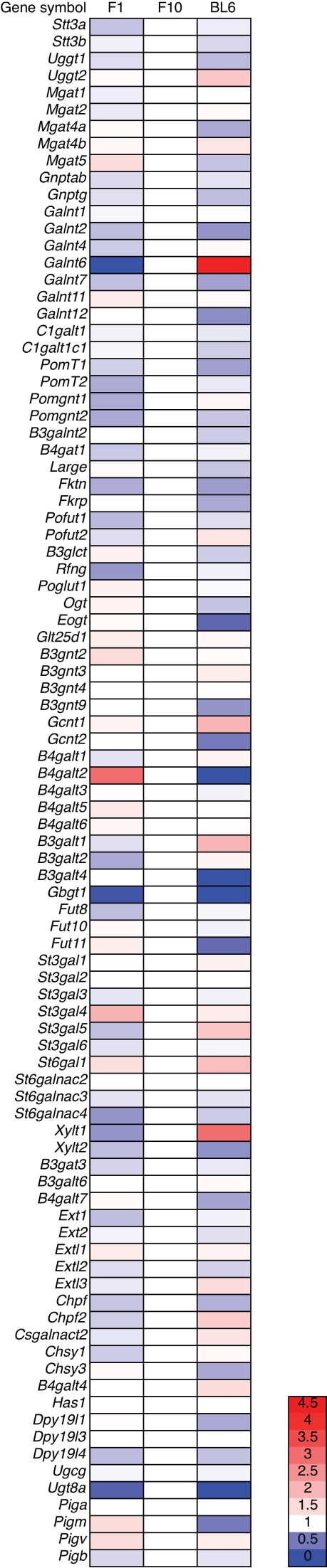

Supplement: Supplementary file 1 — Fig. S1. Relative gene expression levels of 144 glycosyltransferases in B16 variants. [file FEB2-593-942-s001.pdf]

Figure S2

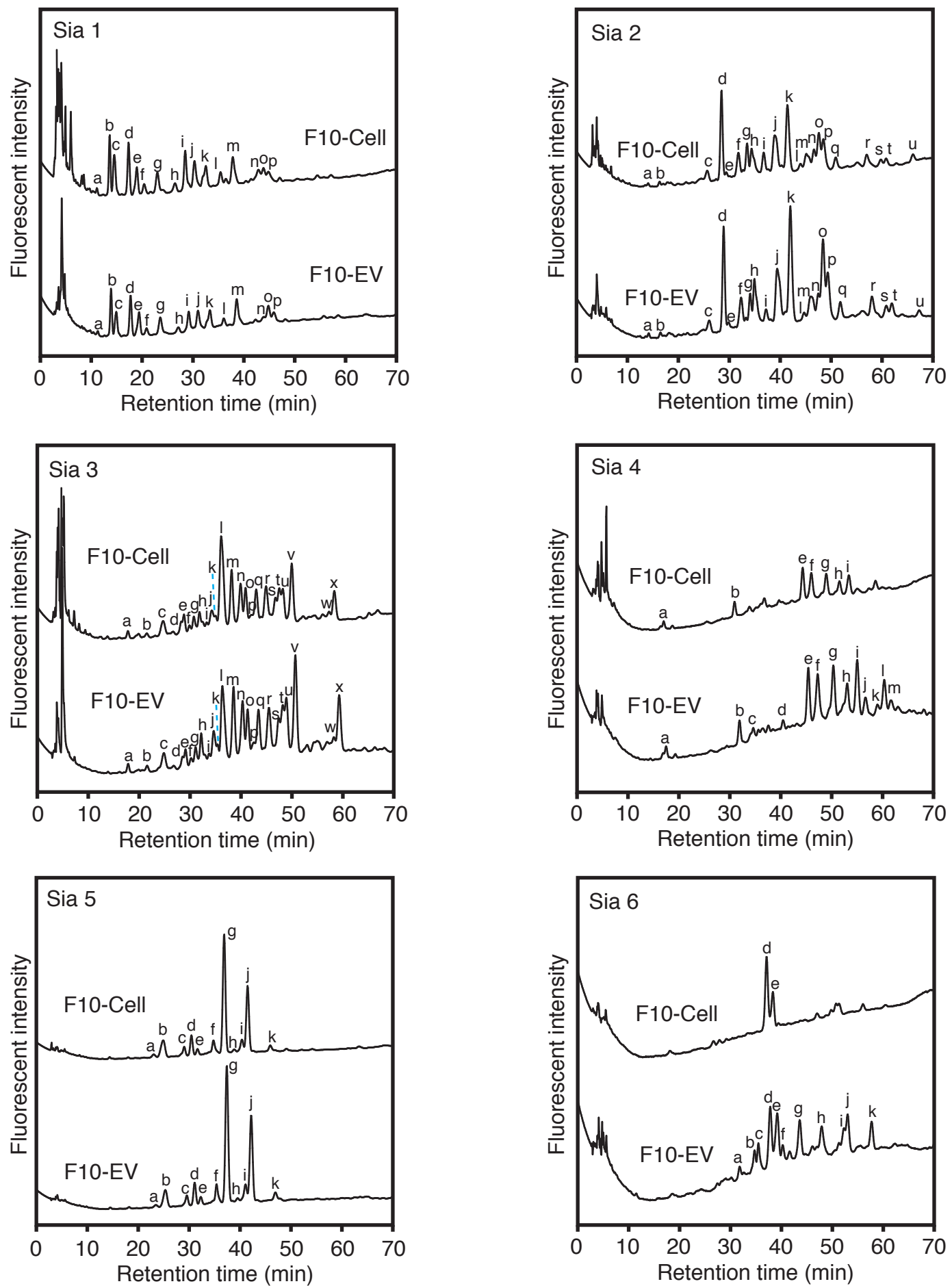

Supplement: Supplementary file 2 — Fig. S2. Comparative analysis of sialylated N‐glycans from B16‐F10 cells and F10‐EVs. [file FEB2-593-942-s002.pdf]

Figure S3 F10-EV F10-Cell

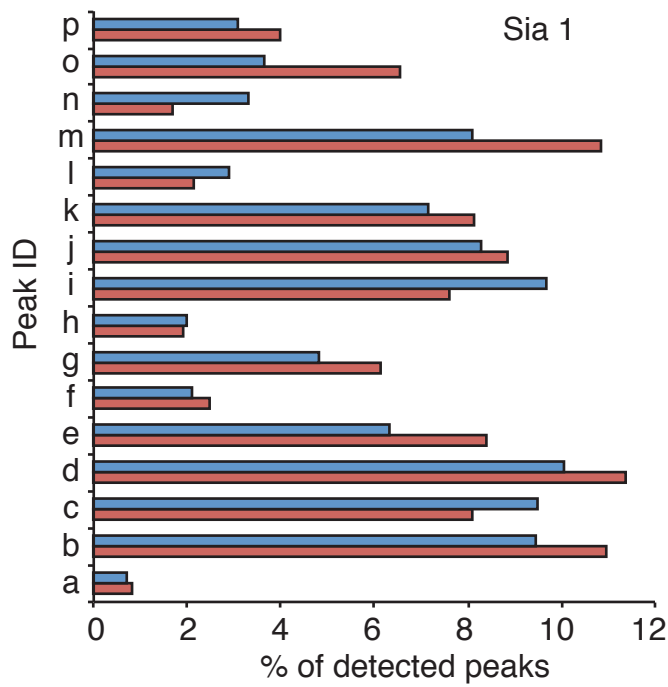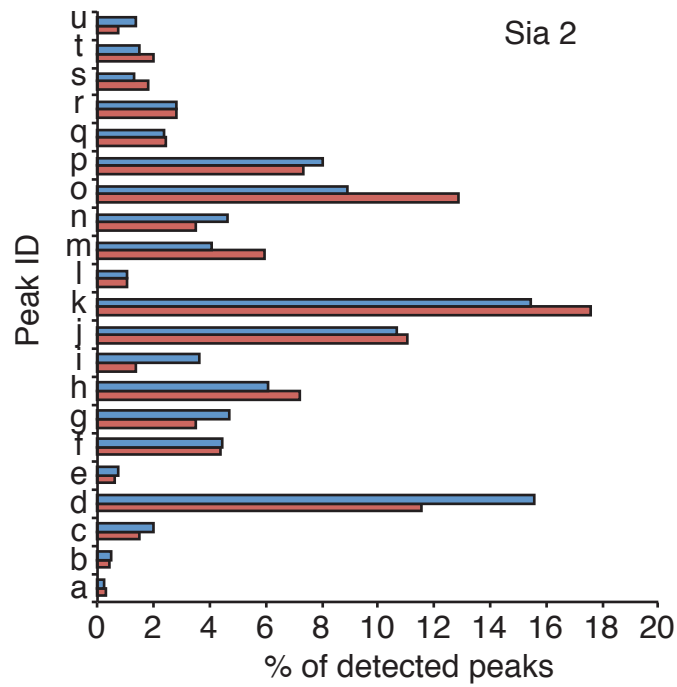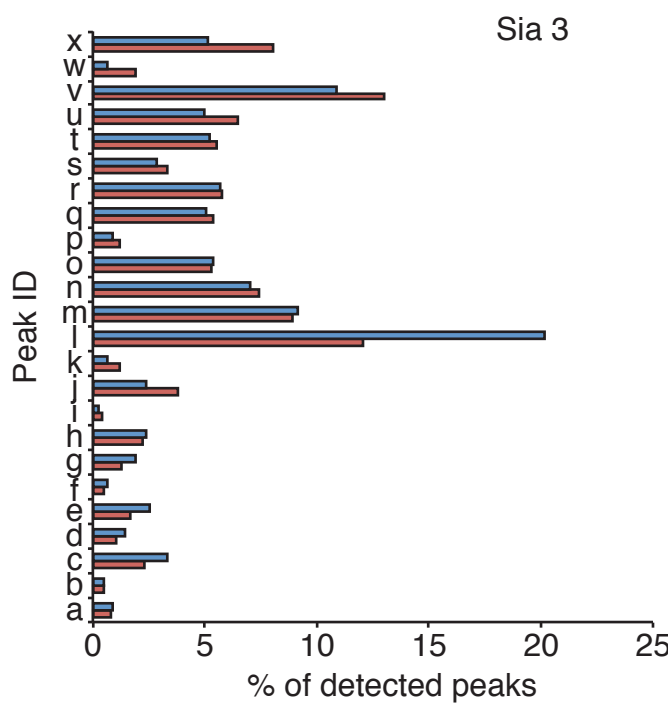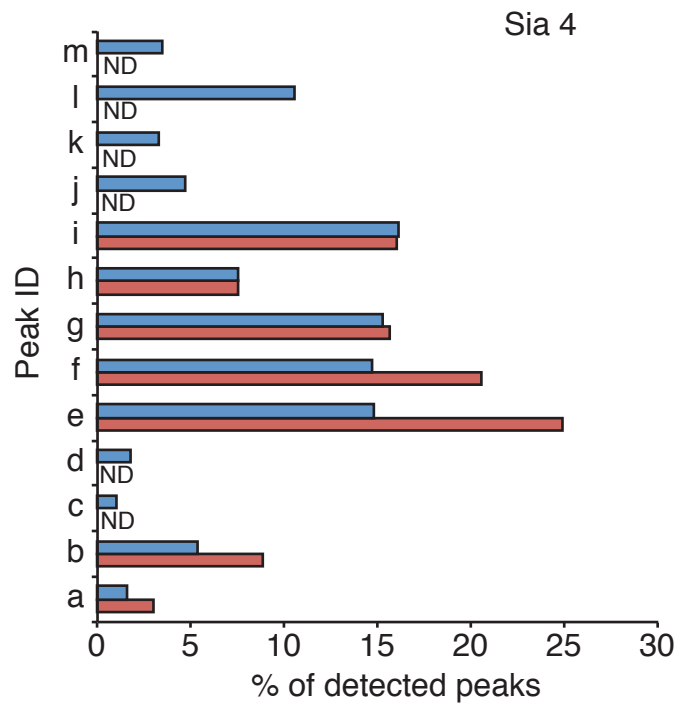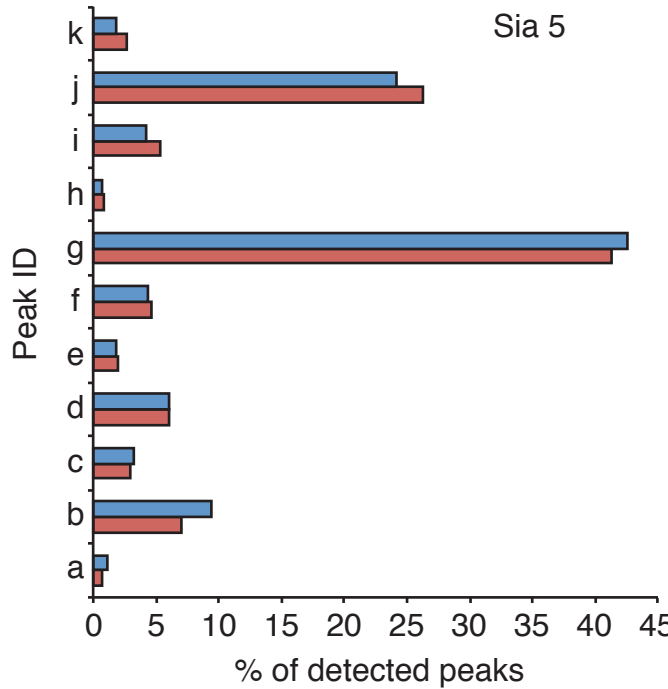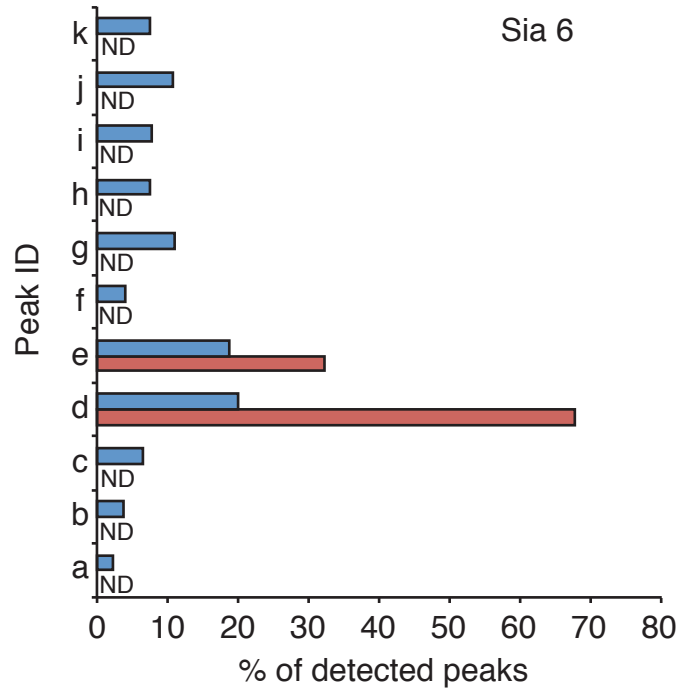

Supplement: Supplementary file 3 — Fig. S3. Relative amounts of sialylated N‐glycans in the Sia 1‐6 fractions of B16‐F10 cells and F10‐EVs. [file FEB2-593-942-s003.pdf]
