# Supplementary material for: N‐glycome inheritance from cells to extracellular vesicles in B16 melanomas
Source: FEBS Lett. 2019 Apr 11;593(9):942–51. doi: 10.1002/1873-3468.13377 (PMC6594130; doi:10.1002/1873-3468.13377)
Supplement: Supplementary file 4 — Table S1. mRNA abundances of glycosyltransferases relative to the mean abundance of four housekeeping genes (Actb, B2m, Gapdh, and Hsp90ab1) in B16 variants. Table S2. Structural analysis of N‐glycans expressed on EVs from B16‐F10 cells. [file FEB2-593-942-s004.docx]

**Supplemental Materials for**

***N*-glycome inheritance from cells to extracellular vesicles in B16 melanomas**

Yoichiro Harada, Yasuhiko Kizuka, Yuko Tokoro, Kiyotaka Kondo, Hirokazu Yagi, Koichi Kato, Hiromasa Inoue, Naoyuki Taniguchi, Ikuro Maruyama

Correspondence to: Yoichiro Harada; [yoharada@m.kufm.kagoshima-u.ac.jp](mailto:yoharada@m.kufm.kagoshima-u.ac.jp)

**The file includes:**

Table S1 and S2

Figs. S1-S3

Supplementary References

**Table S1. mRNA abundances of glycosyltransferases relative to the mean abundance of four housekeeping genes (*Actb*, *B2m*, *Gapdh*, and *Hsp90ab1*) in B16 variants**

|  |  | mRNA abundance | |  |
| --- | --- | --- | --- | --- |
| Gene symbol | Category^a^ | F1 | F10 | BL6 |
| *Stt3a* | *N*-Glycan | 0.418 | 0.543 | 0.505 |
| *Stt3b* | *N*-Glycan | 0.347 | 0.368 | 0.313 |
| *Uggt1* | *N*-Glycan | 0.087 | 0.100 | 0.074 |
| *Uggt2* | *N*-Glycan | 0.017 | 0.016 | 0.028 |
| *Mgat1* | *N*-Glycan | 0.014 | 0.015 | 0.015 |
| *Mgat2* | *N*-Glycan | 0.011 | 0.012 | 0.013 |
| *Mgat3* | *N*-Glycan | ND^b^ | ND^b^ | ND^b^ |
| *Mgat4a* | *N*-Glycan | 0.071 | 0.066 | 0.044 |
| *Mgat4b* | *N*-Glycan | 0.164 | 0.146 | 0.200 |
| *Mgat4c* | *N*-Glycan | ND^b^ | ND^b^ | ND^b^ |
| *Mgat5* | *N*-Glycan | 0.180 | 0.123 | 0.095 |
| *Gnptab* | *N*-Glycan | 0.052 | 0.061 | 0.055 |
| *Gnptg* | *N*-Glycan | 0.014 | 0.016 | 0.012 |
| *Galnt1* | *O*-Glycan | 0.036 | 0.037 | 0.039 |
| *Galnt2* | *O*-Glycan | 0.039 | 0.052 | 0.031 |
| *Galnt3* | *O*-Glycan | ND^b^ | ND^b^ | ND^b^ |
| *Galnt4* | *O*-Glycan | 0.008 | 0.010 | 0.011 |
| *Galnt5* | *O*-Glycan | ND^b^ | ND^b^ | ND^b^ |
| *Galnt6* | *O*-Glycan | ND | 0.018 | 0.077 |
| *Galnt7* | *O*-Glycan | 0.019 | 0.025 | 0.016 |
| *Galnt9* | *O*-Glycan | ND^b^ | ND^b^ | ND^b^ |
| *Galnt10* | *O*-Glycan | ND^b^ | ND^b^ | ND^b^ |
| *Galnt11* | *O*-Glycan | 0.014 | 0.011 | 0.012 |
| *Galnt12* | *O*-Glycan | 0.014 | 0.014 | 0.008 |
| *Galnt13* | *O*-Glycan | ND^b^ | ND^b^ | ND^b^ |
| *Galnt14* | *O*-Glycan | ND^b^ | ND^b^ | ND^b^ |
| *Galnt16(L1)* | *O*-Glycan | ND^b^ | ND^b^ | ND^b^ |
| *Galnt15* | *O*-Glycan | ND^b^ | ND^b^ | ND^b^ |
| *Galntl6(T17)* | *O*-Glycan | ND^b^ | ND^b^ | ND^b^ |
| *Galnt18* | *O*-Glycan | ND^b^ | ND^b^ | ND^b^ |
| *Galntl5(T19)* | *O*-Glycan | ND^b^ | ND^b^ | ND^b^ |
| *Wbscr17* | *O*-Glycan | ND^b^ | ND^b^ | ND^b^ |
| *C1galt1* | *O*-Glycan | 0.066 | 0.069 | 0.063 |
| *C1galt1c1* | *O*-Glycan | 0.025 | 0.026 | 0.021 |
| *PomT1* | *O*-Glycan | 0.009 | 0.011 | 0.007 |
| *PomT2* | *O*-Glycan | 0.017 | 0.025 | 0.023 |
| *Pomgnt1* | *O*-Glycan | 0.040 | 0.059 | 0.067 |
| *Pomgnt2* | *O*-Glycan | 0.006 | 0.009 | 0.007 |
| *B3galnt2* | *O*-Glycan | 0.005 | 0.005 | 0.004 |
| *Mgat5b* | *O*-Glycan | ND^b^ | ND^b^ | ND^b^ |
| *B4gat1* | *O*-Glycan | 0.015 | 0.019 | 0.018 |
| *Large* | *O*-Glycan | 0.015 | 0.014 | 0.011 |
| *Gyltl1b* | *O*-Glycan | ND^b^ | ND^b^ | ND^b^ |
| *Fktn* | *O*-Glycan | 0.022 | 0.032 | 0.020 |
| *Fkrp* | *O*-Glycan | 0.003 | 0.003 | 0.002 |
| *Pofut1* | *O*-Glycan | 0.011 | 0.015 | 0.013 |
| *Pofut2* | *O*-Glycan | 0.027 | 0.031 | 0.042 |
| *B3glct* | *O*-Glycan | 0.018 | 0.015 | 0.012 |
| *Mfng* | *O*-Glycan | ND^b^ | ND^b^ | ND^b^ |
| *Rfng* | *O*-Glycan | 0.011 | 0.018 | 0.017 |
| *Lfng* | *O*-Glycan | ND^b^ | ND^b^ | ND^b^ |
| *Poglut1* | *O*-Glycan | 0.071 | 0.059 | 0.058 |
| *Ogt* | *O*-Glycan | 0.072 | 0.062 | 0.048 |
| *Eogt* | *O*-Glycan | 0.108 | 0.100 | 0.040 |
| *Glt25d1* | *O*-Glycan | 0.088 | 0.070 | 0.077 |
| *Colgalt2* | *O*-Glycan | ND^b^ | ND^b^ | ND^b^ |
| *B3gnt2* | GlcNAc-T | 0.027 | 0.018 | 0.019 |
| *B3gnt3* | GlcNAc-T | 0.004 | 0.004 | 0.005 |
| *B3gnt4* | GlcNAc-T | 0.001 | 0.001 | 0.001 |
| *B3gnt5* | GlcNAc-T | ND^b^ | ND^b^ | ND^b^ |
| *B3gnt6* | GlcNAc-T | ND^b^ | ND^b^ | ND^b^ |
| *B3gnt8* | GlcNAc-T | ND^b^ | ND^b^ | ND^b^ |
| *B3gnt9* | GlcNAc-T | 0.005 | 0.005 | 0.003 |
| *Gcnt1* | GlcNAc-T | 0.007 | 0.006 | 0.012 |
| *Gcnt2* | GlcNAc-T | 0.004 | 0.004 | 0.002 |
| *Gcnt3* | GlcNAc-T | ND^b^ | ND^b^ | ND^b^ |
| *Gcnt4* | GlcNAc-T | ND^b^ | ND^b^ | ND^b^ |
| *A4gnT* | GlcNAc-T | ND^b^ | ND^b^ | ND^b^ |
| *B4galt1* | Gal-T | 0.027 | 0.030 | 0.035 |
| *B4galt2* | Gal-T | 0.003 | 0.001 | ND^b^ |
| *B4galt3* | Gal-T | 0.020 | 0.019 | 0.018 |
| *B4galt5* | Gal-T | 0.060 | 0.046 | 0.046 |
| *B4galt6* | Gal-T | 0.024 | 0.021 | 0.022 |
| *B3galt1* | Gal-T | 0.007 | 0.008 | 0.016 |
| *B3galt2* | Gal-T | 0.004 | 0.006 | 0.007 |
| *B3galt4* | Gal-T | 0.001 | 0.001 | ND^b^ |
| *B3galt5* | Gal-T | ND^b^ | ND^b^ | ND^b^ |
| *A3galt2* | Gal-T | ND^b^ | ND^b^ | ND^b^ |
| *A4galt* | Gal-T | ND^b^ | ND^b^ | ND^b^ |
| *B4galnt1* | GalNAc-T | 0.001 | ND^b^ | ND^b^ |
| *B4galnt2* | GalNAc-T | ND^a^ | ND^b^ | ND^b^ |
| *B4galnt3* | GalNAc-T | ND^a^ | ND^b^ | ND^b^ |
| *B4galnt4* | GalNAc-T | 0.001 | ND^b^ | ND^b^ |
| *Abo* | GalNAc-T | ND^b^ | ND^b^ | ND^b^ |
| *Gbgt1* | GalNAc-T | 0.001 | 0.006 | ND^b^ |
| *Fut1* | Fuc-T | ND^b^ | ND^b^ | ND^b^ |
| *Fut2* | Fuc-T | ND^b^ | ND^b^ | ND^b^ |
| *Fut4* | Fuc-T | ND^b^ | ND^b^ | ND^b^ |
| *Fut7* | Fuc-T | ND^b^ | ND^b^ | ND^b^ |
| *Fut8* | Fuc-T | 0.021 | 0.028 | 0.027 |
| *Fut9* | Fuc-T | ND^b^ | ND^b^ | ND^b^ |
| *Fut10* | Fuc-T | 0.023 | 0.021 | 0.020 |
| *Fut11* | Fuc-T | 0.037 | 0.030 | 0.013 |
| *Sec1* | Fuc-T | ND^b^ | ND^b^ | ND^b^ |
| *St3gal1* | Sia-T | 0.081 | 0.078 | 0.093 |
| *St3gal2* | Sia-T | 0.001 | 0.001 | 0.001 |
| *St3gal3* | Sia-T | 0.019 | 0.021 | 0.020 |
| *St3gal4* | Sia-T | 0.083 | 0.041 | 0.053 |
| *St3gal5* | Sia-T | 0.028 | 0.037 | 0.065 |
| *St3gal6* | Sia-T | 0.235 | 0.263 | 0.254 |
| *St6gal1* | Sia-T | 0.026 | 0.018 | 0.034 |
| *St6gal2* | Sia-T | ND^b^ | ND^b^ | ND^b^ |
| *St6galnac1* | Sia-T | ND^b^ | ND^b^ | ND^b^ |
| *St6galnac2* | Sia-T | 0.001 | 0.001 | 0.001 |
| *St6galnac3* | Sia-T | 0.009 | 0.010 | 0.009 |
| *St6galnac4* | Sia-T | 0.003 | 0.005 | 0.004 |
| *St8sia1* | Sia-T | ND^b^ | ND^b^ | ND^b^ |
| *St8sia3* | Sia-T | ND^b^ | ND^b^ | ND^b^ |
| *St8sia5* | Sia-T | ND^b^ | ND^b^ | ND^b^ |
| *St8sia6* | Sia-T | ND^b^ | ND^b^ | ND^b^ |
| *B3gat1* | HNK-1 | ND^b^ | ND^b^ | ND^b^ |
| *B3gat2* | HNK-1 | ND^b^ | ND^b^ | ND^b^ |
| *St8sia2* | PSA | ND^b^ | ND^b^ | ND^b^ |
| *St8sia4* | PSA | ND^b^ | ND^b^ | ND^b^ |
| *Xylt1* | GAG | 0.003 | 0.005 | 0.015 |
| *Xylt2* | GAG | 0.009 | 0.012 | 0.007 |
| *B3gat3* | GAG | 0.010 | 0.012 | 0.011 |
| *B3galt6* | GAG | 0.012 | 0.012 | 0.013 |
| *B4galt7* | GAG | 0.025 | 0.023 | 0.015 |
| *Ext1* | GAG | 0.015 | 0.020 | 0.019 |
| *Ext2* | GAG | 0.046 | 0.048 | 0.042 |
| *Extl1* | GAG | 0.022 | 0.017 | 0.020 |
| *Extl2* | GAG | 0.020 | 0.023 | 0.019 |
| *Extl3* | GAG | 0.023 | 0.025 | 0.037 |
| *Chpf* | GAG | 0.037 | 0.047 | 0.033 |
| *Chpf2* | GAG | 0.025 | 0.031 | 0.053 |
| *Csgalnact1* | GAG | ND^b^ | ND^b^ | ND^b^ |
| *Csgalnact2* | GAG | 0.019 | 0.021 | 0.029 |
| *Chsy1* | GAG | 0.008 | 0.010 | 0.011 |
| *Chsy3* | GAG | 0.229 | 0.211 | 0.140 |
| *B3gnt7* | GAG | ND^b^ | ND^b^ | ND^b^ |
| *B4galt4* | GAG | 0.014 | 0.014 | 0.021 |
| *Has1* | GAG | 0.001 | 0.001 | 0.001 |
| *Has2* | GAG | ND^b^ | ND^b^ | ND^b^ |
| *Dpy19l1* | *C*-Mannose | 0.050 | 0.048 | 0.032 |
| *Dpy19l2* | *C*-Mannose | ND^b^ | ND^b^ | ND^b^ |
| *Dpy19l3* | *C*-Mannose | 0.005 | 0.005 | 0.005 |
| *Dpy19l4* | *C*-Mannose | 0.032 | 0.043 | 0.033 |
| *Ugcg* | Glycolipid | 0.068 | 0.070 | 0.067 |
| *Ugt8a* | Glycolipid | 0.004 | 0.012 | ND^b^ |
| *Piga* | GPI | 0.002 | 0.002 | 0.002 |
| *Pigm* | GPI | 0.003 | 0.002 | 0.001 |
| *Pigv* | GPI | 0.006 | 0.004 | 0.005 |
| *Pigb* | GPI | 0.016 | 0.019 | 0.017 |

^a^Category [1]: *N*-Glycan, asparagine-linked glycan; *O*-Glycan, serine/threonine-linked glycan; GlcNAc-T, GlcNAc transferase; Gal-T, galactosyltransferase; GalNAc-T, *N*-acetylgalactosaminyltransferase; Fuc-T, fucosyltransferase; Sia-T, sialyltransferase; HNK-1, carbohydrate epitope recognized by HNK-1 antibody; PSA, polysialyltransferase; GAG, glycosaminoglycan; *C*-Mannose, tryptophan *C*-linked mannose; GPI, glycosylphosphatidyl inositol.

^b^ND: Not detected (mRNA abundance less than 0.001).

Table S2. Structural analysis of *N*-glycans expressed on EVs from B16-F10 cells

| Glycan ID^a^ | | GUs in ODS | GUs in Amide | Mass (m/z) | Ion form | GALAXY ID | # of Hex | # of HexNAc | # of dHex |
| --- | --- | --- | --- | --- | --- | --- | --- | --- | --- |
| Neutral | a | 4.8 | 8.8 | 1821.54 | [M+Na]^+^ | M8.1 | 8 | 2 | 0 |
|  | b | 5.1 | 8.0 | 1659.45 | [M+Na]^+^ | M7.2 | 7 | 2 | 0 |
|  |  |  | 9.5 | 1983.52 | [M+Na]^+^ | M9.1 | 9 | 2 | 0 |
|  | c | 5.8 | 7.9 | 1659.41 | [M+Na]^+^ | M7.1 | 7 | 2 | 0 |
|  | d | 6.1 | 7.0 | 1497.32 | [M+Na]^+^ | M6.1 | 6 | 2 | 0 |
|  | e | 7.3 | 6.1 | 1335.40 | [M+Na]^+^ | M5.1 | 5 | 2 | 0 |
| S1 | a | 8.0 | 7.4 | 1700.29 | [M+Na]^+^ | H5.12 | 6 | 3 | 0 |
|  | b | 8.3 | 6.5 | 1538.37 | [M+Na]^+^ | unknown | 5 | 3 | 0 |
|  | c | 10.5 | 7.0 | 1741.40 | [M+Na]^+^ | 200.4 | 5 | 4 | 0 |
|  | d | 10.7 | 7.8 | 1846.45 | [M+Na]^+^ | HF5.12 | 6 | 3 | 1 |
|  |  |  | 9.9 | 2471.65 | [M+Na]^+^ | 400.16 | 7 | 6 | 0 |
|  | e | 11.1 | 7.0 | 1684.36 | [M+Na]^+^ | unknown | 5 | 3 | 1 |
|  | f | 14.6 | 7.4 | 1887.28 | [M+Na]^+^ | 210.4 | 5 | 4 | 1 |
|  | f | 14.6 | 8.2 | 2049.61 | [M+Na]^+^ | unknown | 6 | 4 | 1 |
| S2 | a | 10.5 | 7.0 | 1741.42 | [M+Na]^+^ | 200.4 | 5 | 4 | 0 |
|  | b | 13.4 | 8.4 | 2106.59 | [M+Na]^+^ | 300.8 | 6 | 5 | 0 |
|  | c | 14.6 | 7.4 | 1887.40 | [M+Na]^+^ | 210.4 | 5 | 4 | 1 |
|  |  |  | 10.3 | 2617.62 | [M+Na]^+^ | 410.16 | 7 | 6 | 1 |
|  | d | 18.8 | 8.8 | 2252.52 | [M+Na]^+^ | 310.8 | 6 | 5 | 1 |
| S3 | a | 10.7 | 9.9 | 2471.88 | [M+Na]^+^ | 400.16 | 7 | 6 | 0 |
|  | b | 11.4 | 8.9 | 2252.60 | [M+Na]^+^ | 310.18 | 6 | 5 | 1 |
|  | c | 13.4 | 8.3 | 2106.58 | [M+Na]^+^ | 300.8 | 6 | 5 | 0 |
|  | d | 14.7 | 10.2 | 2618.04 | [M+Na]^+^ | 410.16 | 7 | 6 | 1 |
|  | e | 15.0 | 8.9 | 1887.50 | [M+Na]^+^ | unknown | 5 | 4 | 1 |
|  |  |  | 9.3 | 2252.62 | [M+Na]^+^ | unknown | 6 | 5 | 1 |
|  |  |  | 10.1 | 2455.72 | [M+Na]^+^ | unknown | 6 | 6 | 1 |
|  |  |  |  | 2617.83 | [M+Na]^+^ | unknown | 7 | 6 | 1 |
|  | f | 16.8 | 11.6 | 2983.24 | [M+Na]^+^ | 410.42 | 8 | 7 | 1 |
|  |  |  | 11.4 | 2983.13 | [M+Na]^+^ | unknown | 8 | 7 | 1 |
|  | g | 18.7 | 8.7 | 2252.74 | [M+Na]^+^ | 310.8 | 6 | 5 | 1 |
| S4 | a | 16.8 | 11.6 | 2983.16 | [M+Na]^+^ | 410.42 | 8 | 7 | 1 |
|  | b | 18.1 | 12.4 | 3348.64 | [M+Na]^+^ | 410.52 | 9 | 8 | 1 |
|  | c | 19.2 | 12.4 | 3348.53 | [M+Na]^+^ | unknown | 9 | 8 | 1 |
|  | d | 20.4 | 13.9 | 3713.70 | [M+Na]^+^ | 410.62 | 10 | 9 | 1 |
| S5 | a | 10.7 | 9.9 | 2471.73 | [M+Na]^+^ | 400.16 | 7 | 6 | 0 |
|  | b | 14.7 | 10.2 | 2618.09 | [M+Na]^+^ | 410.16 | 7 | 6 | 1 |
|  | c | 15.1 | 10.1 | 2617.88 | [M+Na]^+^ | unknown | 7 | 6 | 1 |
| S6 | a | 13.9 | 8.2 | 2106.58 | [M+Na]^+^ | 300.22 | 6 | 5 | 0 |
|  | b | 15.0 | 13.7 | 3567.66 | [M+K]^+^ | unknown | 9 | 9 | 1 |
|  | c | 18.0 | 12.9 | 3348.53 | [M+Na]^+^ | 410.52 | 9 | 8 | 1 |
|  | d | 20.3 | 14.0 | 3713.87 | [M+Na]^+^ | 410.62 | 10 | 9 | 1 |

**Supplementary Figure Legends**

**Fig. S1. Relative gene expression levels of 144 glycosyltransferases in B16 variants.** Expression levels of each gene in B16-F10 cells were set to 1.0. Undetectable genes in B16-F10 cells were not shown. The values were calculated as the means of two independent experiments.

**Fig. S2. Comparative analysis of sialylated *N*-glycans from B16-F10 cells and F10-EVs.** Six sialidase-resistant fractions (Sia 1-6; Fig. 2B) from B16-F10 cells (F10-Cell; equivalent to 30 μg protein) and F10-EV (3 μg protein) were analyzed by reversed-phase HPLC. Peaks were detected using the same threshold and minimal peak area throughout the analysis.

**Fig. S3. Relative amounts of sialylated *N*-glycans in the Sia 1-6 fractions of B16-F10 cells and F10-EVs.** Total area of the peaks in each sialidase-resistant fraction was calculated by reversed-phase HPLC and set to 100%. The identity (ID) for each peak was assigned based on Fig. S1. ND, not detected.

**Supplementary References**

1. Kizuka, Y., Nakano, M., Miura, Y. & Taniguchi, N. (2016) Epigenetic regulation of neural N-glycomics, *Proteomics.* **16**, 2854-2863.
